# Theabrownins Produced via Chemical Oxidation of Tea Polyphenols Inhibit Human Lung Cancer Cells *in vivo* and *in vitro* by Suppressing the PI3K/AKT/mTOR Pathway Activation and Promoting Autophagy

**DOI:** 10.3389/fnut.2022.858261

**Published:** 2022-04-21

**Authors:** Yongyong Wang, Yao Yuan, Chunpeng Wang, Bingjie Wang, Wenbin Zou, Ni Zhang, Xiaoqiang Chen

**Affiliations:** ^1^Department of Thoracic Surgery, Tongji Hospital, Tongji Medical College, Huazhong University of Science and Technology, Wuhan, China; ^2^National “111” Center for Cellular Regulation and Molecular Pharmaceutics, Key Laboratory of Fermentation Engineering, Ministry of Education, Hubei University of Technology, Wuhan, China; ^3^Beijing Engineering and Technology Research Center of Food Additives, Beijing Technology and Business University (BTBU), Beijing, China

**Keywords:** theabrownins, chemical oxidation, tea polyphenols, human lung cancer, *in vivo* and *in vitro*

## Abstract

During the fermentation of dark tea, theabrownins (TBs), carbohydrates, and other substances get irreversibly complex. Recent research on the biological activity of TBs is not based on free TBs. In the present study, some brown polyphenol oxidized polymers, the generalized TBs (TBs-C), were prepared via alkali oxidation from tea polyphenols (TP). We also investigated the inhibitory mechanism of TBs-C on non-small-cell-lung cancer (NSCLC). TBs-C demonstrated a stronger inhibition than TP on the NSCLC cell lines A549, H2030, HCC827, H1975, and PC9. Next, A549 and H2030 cell lines were selected as subjects to explore this mechanism. TBs-C was found to inhibit proliferation, promote apoptosis, and induce G1 cell-cycle arrest in the cells. In addition, TBs-C increased autophagic flux, which in turn promoted the death of lung cancer cells. Moreover, TBs-C suppressed the PI3K/AKT/mTOR pathway activation, promoted autophagy, and increased the expression of p21 downstream of AKT, which resulted in G1 cell-cycle arrest. In xenotransplanted NSCLC nude mice derived from A549 cells, TBs-C could significantly suppress tumor growth by inhibiting the PI3K/AKT/mTOR pathway without causing hepatotoxicity, brain toxicity, or nephrotoxicity. We believe that our present findings would facilitate advancement in the research and industrialization of TBs.

## Introduction

Lung cancer is one of the malignant tumors with the highest morbidity and mortality rates worldwide (1) and with an overall 5-year survival rate of <20% (2). Non-small-cell-lung cancer (NSCLC) accounts for approximately 85% of all lung cancers (3). Despite advancements in immunotherapy and targeted therapy, chemotherapy remains the first-line treatment for NSCLC (4, 5). However, exposure to chemotherapies often leads to the development of drug resistance and toxicity, which in turn shortens the progression-free and overall survival times of the patients (4, 6). Therefore, some novel approaches need to be explored toward improving the prognosis of NSCLC.

Autophagy, which is defined as a highly regulated degradation of cytoplasmic macromolecules and organelles in mammalian cells via the lysosomal system (7), serves a housekeeping function by removing misfolded or aggregated proteins, clearing damaged organelles, eliminating intracellular pathogens, and balancing energy sources through critical timepoints during developmental and stress situations (7). Uncontrolled autophagy has been reported to cause a variety of illnesses, such as immunological disorders and cancers (8). However, there is presently no consensus on the relationship between autophagy and cancers, and researchers believe that it plays a dual role in the occurrence and progression of tumors, which depends on the type of tumor, genotypes, and time. Autophagy has emerged as a critical study field in recent years as a target for preventing and treating cancer (9–11). In fact, recent studies have demonstrated that some natural compounds can affect the autophagy of cancer cells, which includes coptisine (12), fisetin (13), and epigallocatechin-3-gallate (14) (EGCG; a major catechin component in green tea).

Natural compounds are receiving increasing attention for their preventive and cure properties in cancer owing to their low toxicity and a variety of bioactivities (11). Tea is one of the world’s most popular non-alcoholic and healthy beverages. Epidemiological studies have associated tea intake with a reduced risk of cancers of the lung, colorectal, liver, and esophageal cancer (15–20). Theabrownins (TBs) are the major bioactive components of dark tea such as Pu’er tea, Chin-brick tea, Fu-brick tea, and Liu Bao tea (21). TBs are water-soluble polyphenol polymers that are oxidized from catechins, and their gallate derivatives are derived throughout the dark tea fermentation process (21).

Numerous studies have indicated that TBs contain a large number of complex heterogeneous components, including carbohydrates, proteins, flavonoids, and other ingredients (22–24). Several harmful organic solvents are used in the separation process of TBs, such as chloroform, ethyl acetate, and n-butanol. In addition, our unpublished research discovered that these heterogeneous components, when combined with TBs, cannot be separated non-destructively despite undergoing several organic solvent extraction processes. Moreover, the preparation process is environmentally hazardous and hence harmful to both humans and animals. These obstacles hinder the optimized utilization and industrialization of TBs. In the past, TBs were isolated from dark tea and their biological activity was investigated. However, in the present study, several brown polyphenols oxidized polymers, that is the generalized TBs (TBs-C), were synthesized from tea polyphenols (TP) through a weak alkali oxidation reaction. The proposed preparation method is eco-friendly and non-toxic. The antitumor effect exerted by TBs-C and its underlying mechanism on NSCLC cells were investigated both *in vivo* and *in vitro* so as to establish a theoretical foundation for its industrialization in the future.

## Materials and Methods

### Preparation of Theabrownins by Weak Alkali Oxidation

Tea polyphenols (purity 98%) were purchased from Taiyo Green Power Co., Ltd. (Jiangsu, China). TBs were prepared from TP by a chemical oxidation assay. NaHCO_3_ was added at a final concentration of 1.00 mg/mL to an aqueous solution containing 8.00 mg/mL TP (purity 95%). The mixture was then heated to 95°C in a water bath for 5 h to initiate oxidative polymerization. The residual NaHCO_3_ and other salt ions in the solution were then removed using an ultrafiltration membrane with a molecular weight cutoff of 1,000 Da to obtain a retentate. Finally, the retentate freeze-dried to obtain TBs (TBs-C). The residual catechin content in TBs-C was measured by HPLC, and the conversion rate of TP oxidized by lye to TBs-C reached 95%. The average weight average molecular weight of TBs-C is 2648 Da, measured by gel permeation chromatography with a multiangle laser light scattering detector and a refractive index detector (GPC-MALLS-RI) (25).

### Cell Lines and Cell Culture

The human lung cancer cell lines A549, H2030, HCC827, H1975 and PC9 were purchased from Cobioer Biosciences (Nanjing, China). Authentication of the A549 cell line was performed using short tandem repeat (STR) DNA profiling at Cobioer Biosciences in 2017. Authentication of the H2030 cell line was performed using STR in 2019. The cells were maintained as a monolayer culture in RPMI 1640 medium supplemented with 10% fetal bovine serum (GIBCO, Grand Island, NY, United States) and 1% penicillin/streptomycin (HyClone, Logan, UT, United States).

### Antibodies and Reagents

The reagents used in this study were as follows: bafilomycin A1 (MedChemExpress, HY-100558), 3-Methyladenine (MedChemExpress, HY-19312), SC 79 (MedChemExpress, HY-18749) and Chloroquine (Enzo Life Sciences 51005-CQC). The antibodies used in this study were as follows: anti LC3A/B (1:1,000, Cell Signaling Technologies, 12741), anti SQSTM1/p62 (1:1,000, Cell Signaling Technologies, 5114), anti cyclin D1 (1:1,000, Cell Signaling Technologies, 55506), anti cyclin E2 (1:1,000, Cell Signaling Technologies, 4132), anti Phospho-Akt (Ser473) (1:1,000 Cell Signaling Technologies, 4060), anti Phospho-PI3 Kinase p85 (Tyr458)/p55 (Tyr199), (1:1,000, Cell Signaling Technologies, 4228), anti Phospho-mTOR (Ser2448) (1:1,000 Cell Signaling Technologies,5536T), anti Akt (1:1,000, Cell Signaling Technologies, 9272), anti p21 Waf1/Cip1 (12D1) (1:1,000, Cell Signaling Technologies, 2947), anti ATG5 (1:5,000, Proteintech Group, 10181-2-AP), anti PI3KR1 (1:5,000, Proteintech Group, 60225-1-Ig), anti mTOR (1:5,000, Proteintech Group, 66888-1-Ig), anti b-actin (1:1,000, Cell Signaling Technologies, 3700), HRP, goat anti mouse IgG polyclone (1:10,000, Abbkine, A21010), HRP, goat anti rabbit IgG polyclone (1:10,000, Abbkine, A21020).

### Colony Formation Assay

For the colony formation assay, 800 cells were seeded into each well in 6-well plates upon the preparation of single-cell suspensions. After 24 h, non-adherent cells were washed away, and adherent cells were cultured for 14 days with various TBs-C concentrations. Every 3 days, the medium was changed. The colonies were fixed with 4% paraformaldehyde, stained with 0.1% crystal violet in 20% methanol for 10 min, washed with PBS and photographed.

### Cell Viability

The vitality of the cells was determined using the Cell Counting Kit-8 (CCK-8 kit, Dojindo Molecular Laboratories, Kumamoto, Japan). Before exposure to the relevant medicines, cell suspensions were seeded (3,000∼5,000 cells/100 μL/well) and cultured overnight. The absorbance was determined using a microplate spectrophotometer at a wavelength of 450 nm (TECAN).

### Cell Cycle Analysis

The cells (1 × 10^5^) were labeled with propidium iodide (PI)/RNase Staining Buffer (BD Biosciences, San Jose, CA, United States) for cell cycle analysis. The samples were analyzed using an Attune NxT Acoustic cytometer (Thermo Fisher Scientific, United States) and all data were analyzed using ModFit software (Verity Software House, Inc., Topsham, ME, United States).

### Cell Apoptosis Analysis

Cells treated with or without TBs-C were collected and washed twice with PBS. Subsequently, the cells were stained with an Annexin V/7-AAD Apoptosis Detection Kit (BD Biosciences, San Jose, CA, United States). The samples were analyzed using an Attune NxT Acoustic cytometer (Thermo Fisher Scientific, United States) within 1 h of processing. The percentage of cells in each quadrant of the dot plot was determined using FlowJo v10 (BD Biosciences).

### Western Blot Analysis

All cells were lysed with RIPA lysis buffer. Cell lysates were resolved by SDS-PAGE and transferred onto PVDF membranes. The blots were developed using an enhanced chemiluminescent detection system (Tanon 5200, Shanghai, China). To ensure that equal amounts of sample proteins were applied to each lane, β-actin was used as the loading control.

### Autophagosome Staining

Cells were seeded on coverslips and incubated for 30 min at 37°C with 2 L/mL CYTO-ID Green detection reagent (Enzo Life Sciences, Farmingdale, NY, United States). The nucleus was counterstained with Hoechst 33,342 staining. A wide-field fluorescence microscope (Carl Zeiss, Baden-Württemberg, Germany) was used to take images immediately after staining. The area of the CYTO-ID green puncta was determined using the FIJI plugin Analyze Particle. We analyzed three randomly selected images from each group.

### Tandem StubRFP-SensGFP-LC3B Fluorescence Assay

A stubRFP-sensGFP-LC3B lentiviral expression vector was purchased from GENE (Shanghai, China). Cells were transfected with the stubRFP-sensGFP-LC3B lentiviral expression vector following the instructions. The transfected cells were screened with 2 μg/mL puromycin (Beyotime) until almost all cells expressed fluorescence. Stable cell lines were treated with or without TBs-C, followed by fixation with 4% paraformaldehyde and nuclear staining with DAPI. The cells were visualized using a wide-field fluorescence microscope (Carl Zeiss, Baden-Württemberg, Germany). We quantified the numbers of red and yellow puncta using the Analyze Particle plugin for FIJI. A hue of 0–20 was counted as a red point. A hue of 20–255 was counted as a yellow point.

### siRNA Transfection

Short interfering RNA (siRNA) sequences specifically targeting human lung cancer cells ATG5 were synthesized by RiboBio (Guangzhou, China). Reverse transfection was used to deliver siRNA into cells. Briefly, siRNA (50 nM) and Lipofectamine 3000 (Invitrogen) were gently premixed in medium without serum according to the manufacturer’s recommendations. After adding the transfection mixture to the culture plate, cell suspensions were seeded into the culture plate and maintained in 10% FBS for 48 h. The efficacy of ATG5 knockdown was assessed by Western blot analysis.

### Animal Treatment

All animal experiments were approved by the Animal Care Committee of Tongji Hospital, Huazhong University of Science and Technology. A total of 18 female, SPF grade BALB/c nude mice, aged 5 weeks and weighing 16–18 g, were purchased from Beijing Weitong Lihua Experimental Animal Technology Co. The experimental mice were inoculated with 1 × 10^6^ A549 tumor cells in the right armpit. When the tumor grew to an average volume of 100 mm^3^, the mice were randomly divided into three groups according to the tumor size and body weight (*n* = 6 in each group) including the control group (oral administration of saline), low-dose group (oral administration of TBs-C 150 mg/kg⋅d) and high-dose group (oral administration of TBs-C 300 mg/kg⋅d). Tumor size and body weight were measured twice per week. The tumor volume was calculated using the following formula: tumor volume (mm^3^) = (short diameter)^2^ × (long diameter) × 0.5. After oral administration for 28 days, the mice were killed humanely under anesthesia and photographed, and then the tumors were excised, weighed, and photographed. The heart, kidney, and brain were further observed by H&E staining using an optical microscope. Western blotting was performed on the tumor tissues obtained from four mice randomly selected from each group and β-actin was used as a loading control.

### Statistical Analysis

We performed statistical analyses by using Prism V8.0 software (GraphPad, La Jolla, CA, United States). Data in the bar graphs are presented as the mean ± SEM. A two-sided Student’s *t* test was used for two groups, and ANOVA with Dunnett’s multiple comparisons test was used for more than two groups. A *p* value < 0.05 was considered statistically significant.

## Results

### Comparison of the Anti-tumor Effects of TBs-C and Tea Polyphenols on Human Non-small-cell-lung Cancer Cells

**FIGURE 1:**
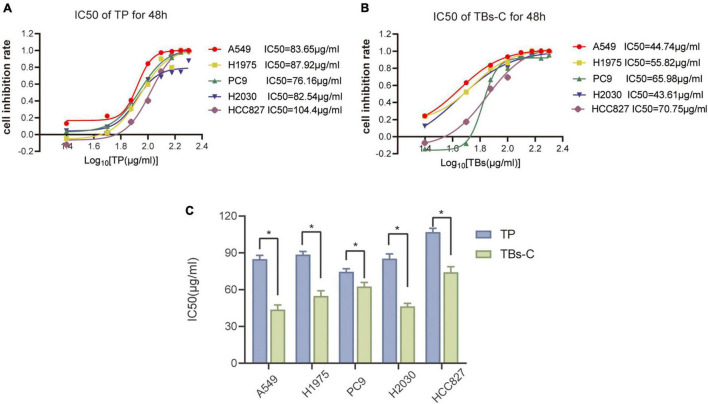
Comparison of the anti-tumor effects of TBs-C and TP on NSCLC cells. **(A,B)** The experimental cells were treated with different concentrations (0, 25, 50, 75, 100, 125, 150, 175, and 200 μg/mL) of TBs-C and TP for 48 h, and the cell viability was determined by the CCK-8 assay, with the calculation of the IC50 values. **(C)** The comparison of the IC50 values between TP and TBs-C in different NSCLC cell lines are presented, *p < 0.05.

Several studies have shown that TP exhibit anticancer activity against many cancer cells, including lung cancer (18, 26, 27). The antitumor effects of TBs-C and TP were compared in various cell lines (A549, HCC827, H2030, PC9, and H1975). The CCK-8 assay was used to determine the inhibitory effects of TP and TBs-C (concentrations of 0, 25, 50, 75, 100, 125, 150, 175, and 200 g/mL), and IC50 curves were constructed (Figures 1A,B). The results of the comparison (Figure 1C) demonstrated that TBs-C had a lower IC50 value than TP, thus indicating that TBs-C is more effective in inhibiting lung cancer than TP *in vitro*.

### Effects of TBs-C on Lung Cancer Cell Proliferation, Apoptosis, and Cell Cycle *in vitro*

**FIGURE 2:**
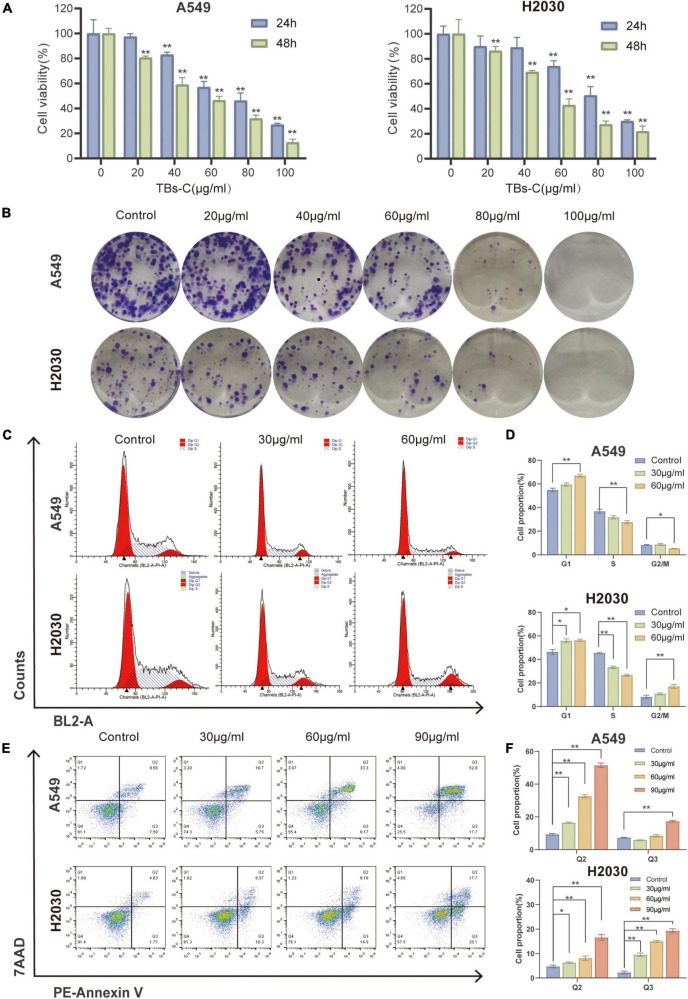
Effects of TBs-C on lung cancer cell proliferation, apoptosis, and cell cycle *in vitro*. **(A)** The CCK-8 assay was performed to determine the viability of A549 and H2030 cell lines after 24 and 48 h of treatment with different doses of TBs-C. **(B)** The capacity of A549 and H2030 cell lines to form clones was examined at varying concentrations of TBs-C. **(C)** The analysis of the cell cycle by flow cytometry after a 24 h treatment with TBs-C is depicted. **(D)** Quantitative results of the cell cycle analysis. **(E)** Evaluation of the apoptosis of cells treated for 24 h of TBs-C by AnnexinV-7AAD flow cytometry. **(F)** Quantitative analysis of cell apoptosis, *p < 0.05, **p < 0.01.

To investigate the effects of TBs-C on lung cancer cells, two human NSCLC cell lines, namely A549 and H2030, were chosen. The CCK-8 assay was employed to assess cell viability after 24 or 48 h of treatment with various concentrations of TBs-C (20, 40, 60, 80, and 100 g/mL). The results (Figure 2A) revealed that TBs-C inhibited the proliferation of NSCLC cells in a dose- and time-dependent manner. Moreover, when the TBs-C concentration was increased, there was a significant decrease in the number of clones formed by A549 and H2030 cells (Figure 2B), which confirmed the inhibitory effect of TBs-C on the proliferation of lung cancer cell lines.

Owing to the crucial role of the cell cycle in tumor cell proliferation, the cells were treated with various doses of TBs-C and the cell cycle was analyzed using flow cytometry. As shown in Figures 2C,D, TBs-C treatment significantly increased the proportion of A549 and H2030 cells in the G1 phase. On the contrary, this treatment decreased the proportion of cells in the S phase, which suggested that TBs-C significantly affected the cell cycle and triggered cell cycle arrest in the G1 phase.

Apoptosis, a type of programmed cell death, is a significant mechanism of anticancer drug-induced cell death. To detect cell apoptosis caused by TBs-C, flow cytometry studies were performed with annexin V/7-AAD double labeling. The results indicated that TBs-C tended to significantly increase the proportion of apoptotic lung cancer cells (both early and late apoptosis) (Figures 2E,F). According to the data, TBs-C inhibited proliferation, induced G1 phase arrest, and promoted apoptosis in NSCLC cells *in vitro*.

### Accumulation of Autophagosomes in TBs-C-Treated Lung Cancer Cells

Autophagy is a highly conserved catabolic process in which large intracellular components, such as long-lived proteins and dysfunctional organelles, are engulfed by double-membrane vesicles termed autophagosomes and then degraded via the lysosomal pathway (7). Autophagy is an important physiological function that promotes cell survival and regulates cell death dynamics and is, therefore, a critical target for cancer therapy (28). Hence, the ability of TBs-C to impair autophagy in human lung cancer cells was explored. LC3 has previously been used to monitor autophagy, and the level of LC3-II is correlated with the number of autophagosomes. p62, a substrate adapter and a key regulator of autophagy, is similarly degraded upon autophagy activation, thereby making it another widely employed reporter for cellular autophagy activity.

**FIGURE 3:**
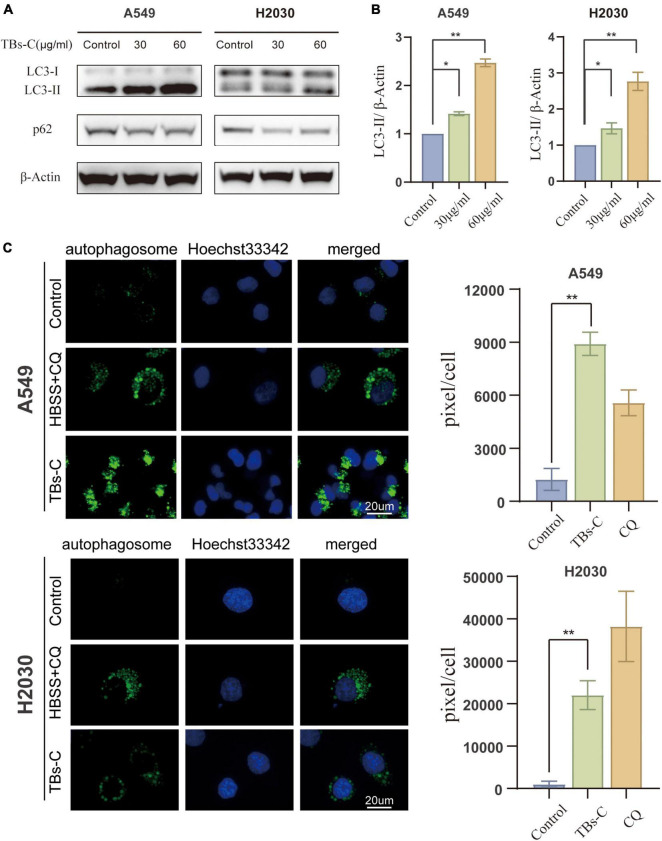
Accumulation of autophagosomes in TBs-C treated lung cancer cells. **(A)** Western blotting results of the LC3 and p62 protein expression in TBs-C treated and untreated cells. **(B)** The densitometric analysis of the changes in the abundance of LC3-II normalized to the β-actin level. **(C)** The indicated cells were treated with TBs-C (60 μg/mL) or untreated for 24 h. The cells were labeled with CYTO-ID^®^ green detection reagent at the end of the treatment period to detect the autophagosomes. As a positive control for autophagosome accumulation, the cells were treated with HBSS in the presence of CQ (60 M) for 3 h. The puncta areas per cell are given in three randomly selected images for each culture condition, *p < 0.05, **p < 0.01.

Treatment of NSCLC cells with TBs-C increased the levels of LC3-II and decreased the levels of p62 (Figures 3A,B). The CYTO-ID green detection reagent was utilized in this study to confirm the findings of western blotting because it selectively stains the autophagic vesicles. Accumulation of autophagosomes was observed in cells treated with TBs-C and HBSS + chloroquine (CQ) (Figure 3C). These findings imply that TBs-C therapy can cause the accumulation of autophagosomes in human lung cancer cells.

### Treatment With TBs-C Promotes Autophagic Flux in Non-small-cell-lung Cancer Cells

**FIGURE 4:**
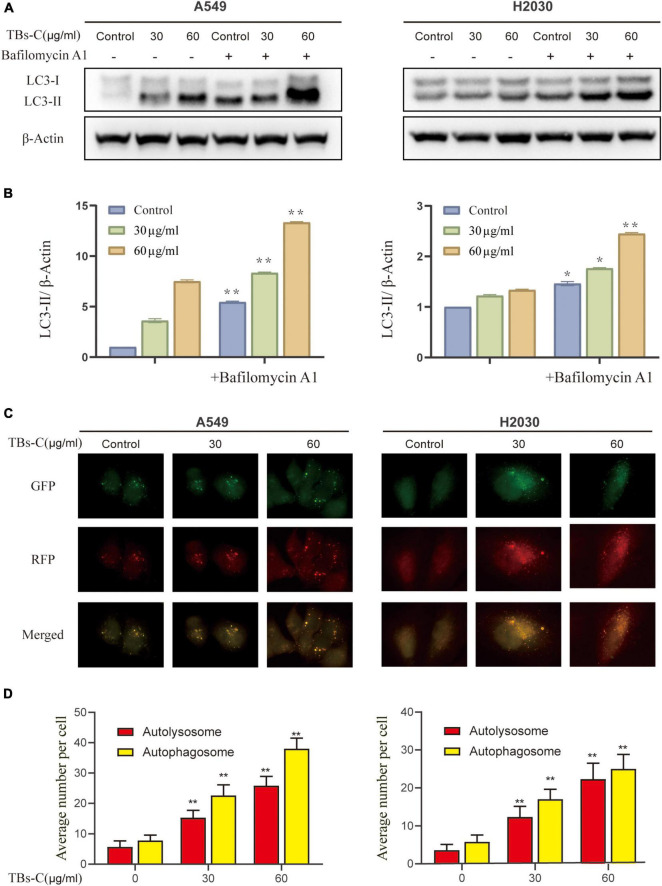
Treatment with TBs-C promotes the autophagic flux in NSCLC cells. **(A)** After 24 h of treatment with different concentrations of TBs-C, the cells were treated with or without baf A1 (100 nmol/mL for 3 h), and the LC3 expression was evaluated by Western blotting. **(B)** Densitometric analysis of the changes in LC3-II abundance normalized to the β-actin level. **(C,D)** The indicated cells were transfected with the RFP-GFP-LC3B Premo™ Autophagy Tandem Sensor. Fluorescence microscopy was used to count the red and yellow puncta (Scale bar = 20 m). The number of yellow and red puncta in each cell was calculated from at least 20 cells from each group, *p < 0.05, ^**^p < 0.01.

Autophagy is initiated mechanically by the formation of double-membrane vesicles termed autophagosomes, which subsequently capture the cytosolic cargo and deliver it to the lysosomes, where the products are degraded and eventually recycled back to the cytoplasm. This dynamic process is known as autophagic flux (29). Autophagosome accumulation could occur because of an increase in its formation or a decrease in its degradation (30). To further examine these two possibilities, the effect of TBs-C on the autophagic flux of NSCLC cells was examined. The ratio of LC3-II to β-actin was determined in cells treated with various doses of TBs-C with or without the autophagosome–lysosome fusion inhibitor bafilomycin A1 (Baf A1). As demonstrated in Figures 4A,B, cells treated with TBs-C had a higher level of LC3-II than those not treated with TBs-C. Additionally, cells treated with TBs-C in combination with Baf A1 accumulated more LC3-II than those treated with TBs-C or Baf A1 individually. It has been proposed that TBs-C may promote the development of autophagosomes in the pre-autophagic state.

To confirm the effect of TBs-C on autophagic flow, cells were transfected with the Premo^^TM^ Autophagy Tandem Sensor RFP-GFP-LC3B. It was found that while the GFP signaling was quenched in the acidic autolysosomes, the RFP signaling remained stable. The colocalization of GFP and RFP suggests that the autophagosome is not fused with the lysosome. In contrast, the RFP signal in the absence of GFP indicates the presence of an autolysosome. This method of estimating the autophagic flux is considered to be more sensitive and precise than western blotting (29). As shown in Figures 4C,D, the fluorescence microscopy images revealed a significant increase in the red and yellow spots following the addition of TBs-C, which signified a simultaneous increase in autophagosomes and autophagic lysosomes. These findings confirm that TBs-C treatment promotes autophagic flux in NSCLC cells.

### Inhibition of Autophagy Overcomes the Antitumor Effect of TBs-C in Non-small-cell-lung Cancer Cells

**FIGURE 5:**
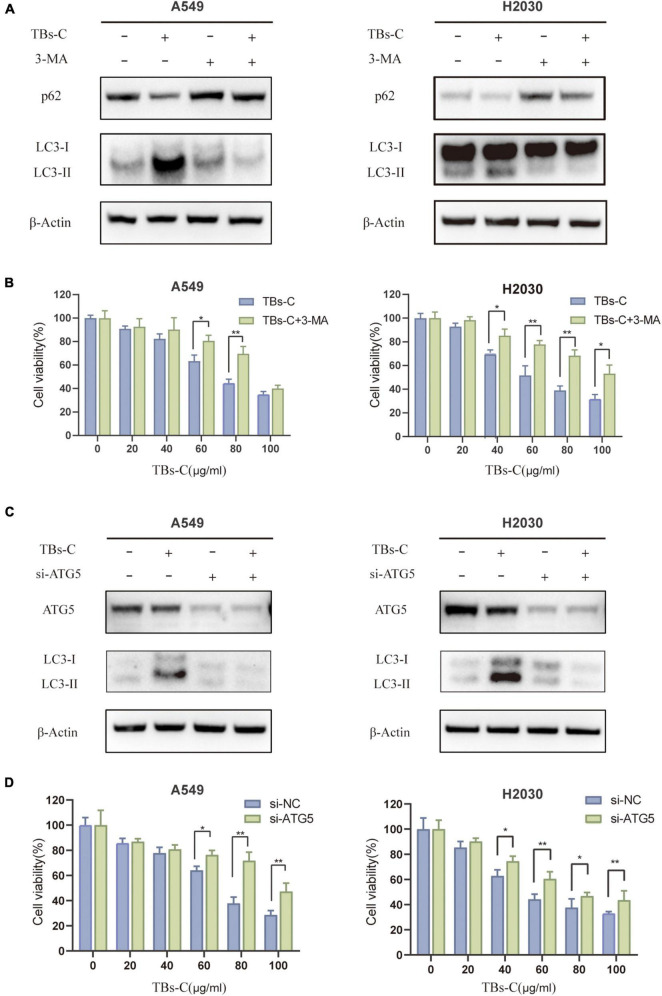
Inhibition of autophagy overcomes the antitumor effect of TBs-C in NSCLC cells. **(A)** Cells pretreated with or without 3-MA were treated with TBs-C (60 μg/mL) for 24 h, and the protein levels of LC3 and p62 were detected by Western blotting. **(B)** Cells pretreated with or without 3-MA were treated with TBs-C for 24 h, and the cell viabilities were determined by the CCK-8 assay. **(C)** Cells transfected with si-NC and si-ATG5 were treated with TBs-C (60 μg/mL) for 24 h, and the protein levels of LC3 and p62 were detected by Western blotting. **(D)** Cells transfected with si-NC and si-ATG5 were treated with TBs-C for 24 h, and the cell viabilities were determined by CCK-8 assay, *p < 0.05, **p < 0.01.

Autophagy functions as a double-edged sword in cancer because it can either promote or inhibit disease progression (28). Autophagy dysfunction is associated with an increased risk of cancer progression because of DNA damage accumulation and genomic instability. For example, the incidence of Kras-driven lung cancer is significantly increased in ATG5-deficient mice (31). On the contrary, autophagy has been shown to be upregulated in many types of established tumors as a tumor survival mechanism, for example, to meet the high metabolic requirements of the tumor cells (32). The effect of TBs-C-induced autophagy enhancement on lung cancer cells was investigated. The changes in the expressions of LC3 and p62 as well as cell viability in lung cancer cells treated with or without 3-MA (an upstream autophagy inhibitor) before TBs-C treatment were assessed. As illustrated in Figures 5A,B, the expression of LC3-II was reduced considerably in cells treated with 3-MA compared with the untreated group, whereas the level of p62 was increased, thereby suggesting that 3-MA inhibited the autophagy triggered by TBs-C in lung cancer cells. The results of cell viability indicated that TBs-C had a significantly lower inhibitory effect on 3-MA-pretreated cells than the control group, thus showing that TBs-C-induced autophagy may suppress the proliferation of lung cancer cells.

To understand whether TBs-C-induced autophagy leads to cell death in human lung cancer cells, A549 and H2030 cells were transfected with Atg5 siRNA to knockdown Atg5 expression. ATG5 is a key ATG protein that is involved in autophagy by forming a complex with ATG12 that promotes the transformation of LC3-II and autophagy precursors (33). As seen in Figures 5C,D, after knocking down the expression of ATG5, the level of LC3-II decreased in the cells, which implied the inhibition of autophagy. ATG5 knockdown considerably reduced the inhibitory effect of TBs-C on lung cancer cells. In summary, TBs-C promotes the death of lung cancer cells and inhibits their proliferation by enhancing autophagy.

### TBs-C Inhibits the PI3K/AKT/mTOR Pathway in Non-small-cell-lung Cancer Cells

**FIGURE 6:**
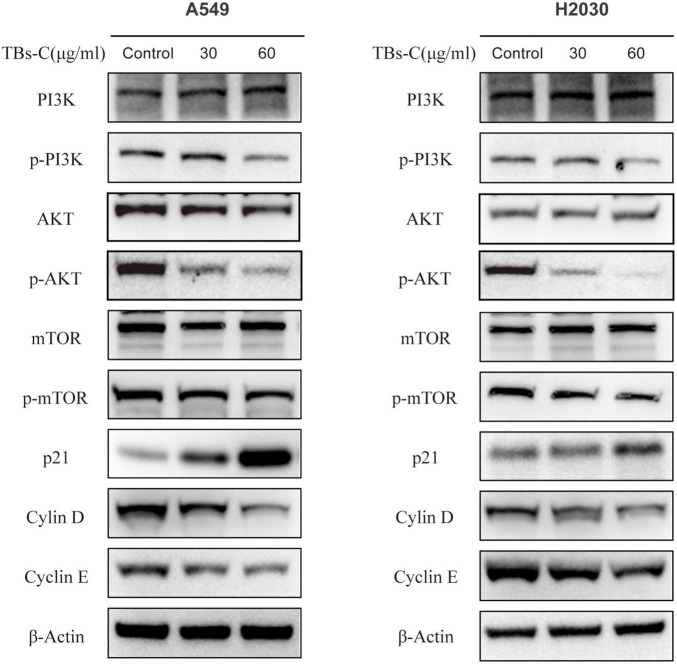
TBs-C inhibits the PI3K/AKT/mTOR pathway in NSCLC cells. The expression of major proteins in the PI3K/AKT/mTOR pathway and the AKT/p21 signal axis was detected by Western blotting in cells treated with different concentrations of TBs-C, with β-actin was used as a reference.

Autophagy is a complex biological process that is regulated via multiple pathways. The phosphoinositide 3-kinase (PI3K)/protein kinase B (AKT)/mammalian target of rapamycin (mTOR) signaling pathway is critical in autophagy and is closely associated with cell cycle, apoptosis, proliferation, and metabolism (34). It was hypothesized that TBs-C induces autophagy by inactivating the PI3K/AKT/mTOR signaling pathway. To prove this hypothesis, the expressions of proteins involved in the pathway were detected using western blot analysis. In A549 and H2030 cells treated with TBs-C, the levels of phosphorylated PI3K, AKT, and mTOR decreased significantly in comparison with the control group. However, the total PI3K, Akt, and mTOR expression levels were similar between the two groups (Figure 6). These findings suggest that TBs-C suppresses the activation of the PI3K/AKT/mTOR pathway.

p21 (Waf1/Cip1), a cyclin-dependent kinase 1A (CDKN1A) inhibitor, is one of the major downstream target genes of AKT and is essential for the cell cycle (35). p21 inhibits the activity of the cyclin D-CDK4 and cyclin E-CDK2 complexes. Thus, the cells are prevented from entering the S phase from the G1 phase, which constitutes the chief mechanism by which the G1–S transition is blocked (36). After confirming that TBs-C treatment suppressed the level of phosphorylated AKT, the expressions of p21 and its downstream molecules were detected. As shown in Figure 6, p21 expression increased, whereas cyclin D and cyclin E expressions decreased in the TBs-C treated cells. These results allude that TBs-C may promote autophagy by suppressing the PI3K/AKT/mTOR pathway and increasing p21 expression via the AKT/p21 signaling axis, which results in the G1-phase arrest of human lung cancer cells.

### TBs-C Induces Autophagy and G1 Cell Cycle Arrest in Non-small-cell-lung Cancer Cells by Inhibiting the PI3K/AKT/mTOR Pathway

**FIGURE 7:**
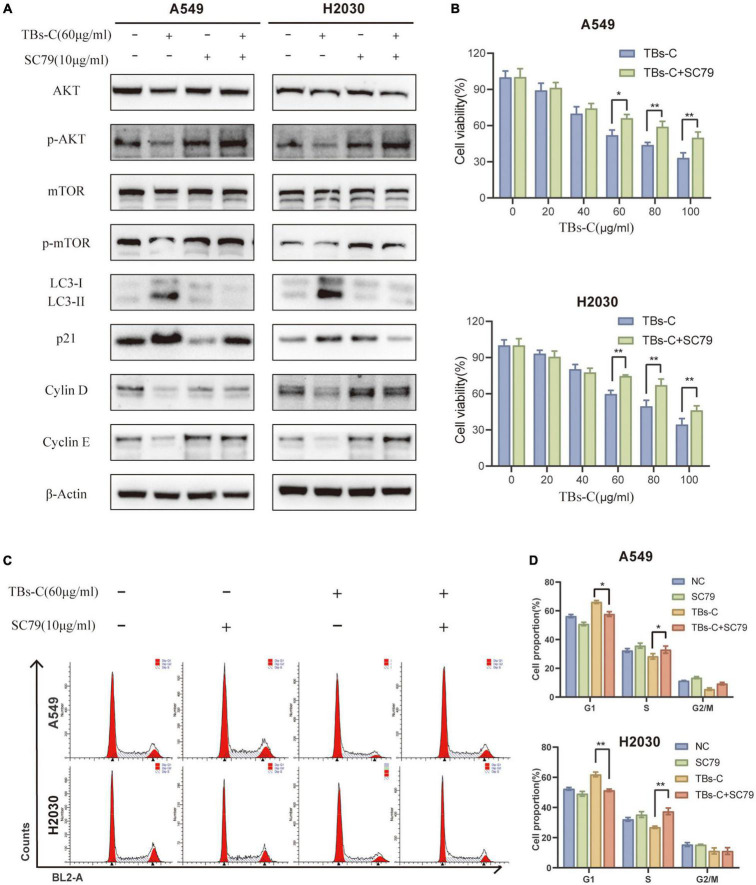
TBs-C induces autophagy and the G1 cell-cycle arrest in NSCLC cells by inhibiting the PI3K/AKT/mTOR pathway. **(A)** The expression of AKT, p-AKT, mTOR, p-mTOR, p21, LC3, cyclin D, and cyclin E in the indicated cells was detected by Western blotting. **(B)** The cell viability of indicated cells were measured by the CCK-8 assay. **(C)** The cell cycle distribution of each group was determined by flow cytometry. **(D)** Analysis of cell cycle distribution by histogram, *p < 0.05, **p < 0.01.

To establish whether TBs-C promotes autophagy and induces G1 phase arrest in lung cancer cells by inhibiting the PI3K/AKT/mTOR pathway, the cells were treated with TBs-C (60 μg/mL) after pretreatment with the AKT activator SC79 (10 μg/mL). The related protein expression was determined using western blotting. The results (Figure 7A) suggested that the combined treatment with SC79 and TBs-C did not cause any significant change in the levels of total AKT and mTOR; however, the levels of p-AKT and p-mTOR were increased and the level of p21 was decreased, which denoted that SC79 partially reversed the effects of TBs-C on the PI3K/AKT/mTOR and AKT/p21 signaling pathways. Importantly, LC3-II expression was increased in cells treated with SC79 and TBs-C compared with those treated with TBs-C alone, whereas cyclin D and cyclin E expressions were decreased, which meant that TBS-C-induced G1 cell cycle arrest and autophagy were suppressed. Furthermore, the changes in cell viability and cell cycle in the aforementioned experimental groups were analyzed using a CCK-8 assay and flow cytometry. The results (Figures 7B–D) indicated that SC79 can partially reverse the suppression of cell viability and G1 arrest caused by TBS-C in NSCLC cells. Based on the findings, it was concluded that TBs-C enhances autophagy and induces G1 phase arrest in human lung cancer cells predominantly by inhibiting the PI3K/AKT/mTOR pathway.

### TBs-C Inhibits the Growth of A549 Xenograft Tumors *in vivo*

**FIGURE 8:**
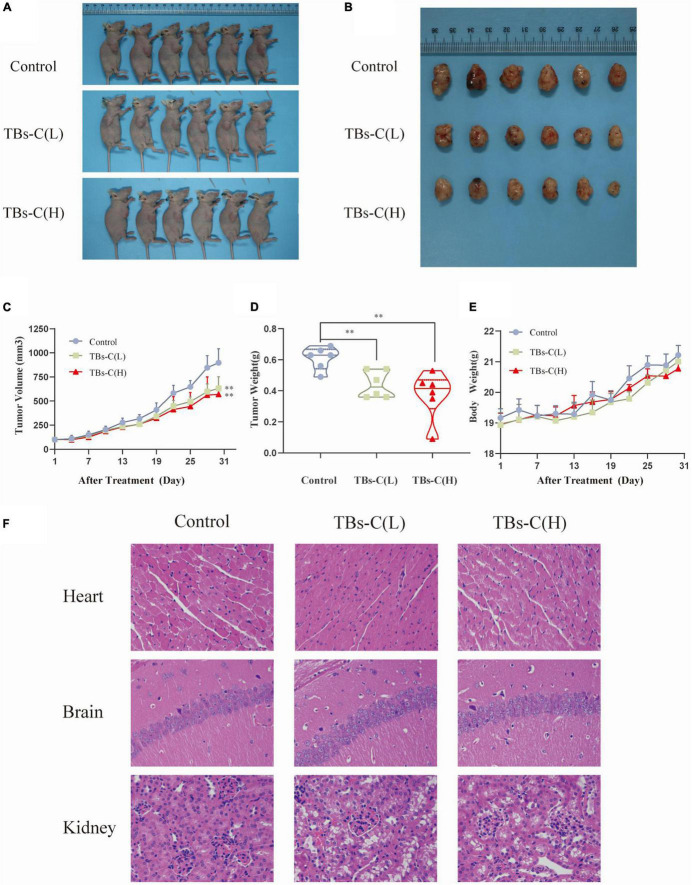
TBs-C inhibits the growth of A549 xenograft tumors *in vivo*. **(A)** Tumorigenesis of A549 cells of the 3 study groups on day 28. **(B)** Representative size of the 3 groups of tumors. **(C)** Tumor volumes (measured every 3 days). **(D)** Tumor weight in the control group, low-dose group, and high-dose group. **(E)** The weight of nude mice was recorded every 3 days. **(F)** H&E stained the heart, brain, and kidney of mice from each group. TBs-C (L), TBs-C low-dose group, TBs-C (H), TBs-C high-dose group, *p < 0.05, **p < 0.01.

Based on the above-mentioned *in vitro* findings, a human NSCLC A549 cell xenograft model was created to investigate the antitumor effect of TBs-C in BALB/c nude mice. After 28 days of oral administration, the average tumor volume and weight in the low-dose TBs-C (150 mg/kg/day) and high-dose TBs-C (300 mg/kg/day) treatment groups decreased significantly in comparison with the control group (Figures 8A–D). This result implied that TBs-C can also inhibit the growth of NSCLC tumors *in vivo*. Moreover, the toxicity of TBs-C was studied. No significant reduction was observed in body weight in either the high- or low-dose groups compared with the control group (Figure 8E). The hearts, brains, and kidneys of the mice in each group were subjected to H&E staining and histological examination. The results (Figure 8F) signified that when the mice were administered a dose of 150–300 mg/kg/day, no significant pathological abnormalities occurred in their major organs (heart, brain, and kidney). Collectively, these findings establish that the oral administration of TBs-C is safe.

**FIGURE 9:**
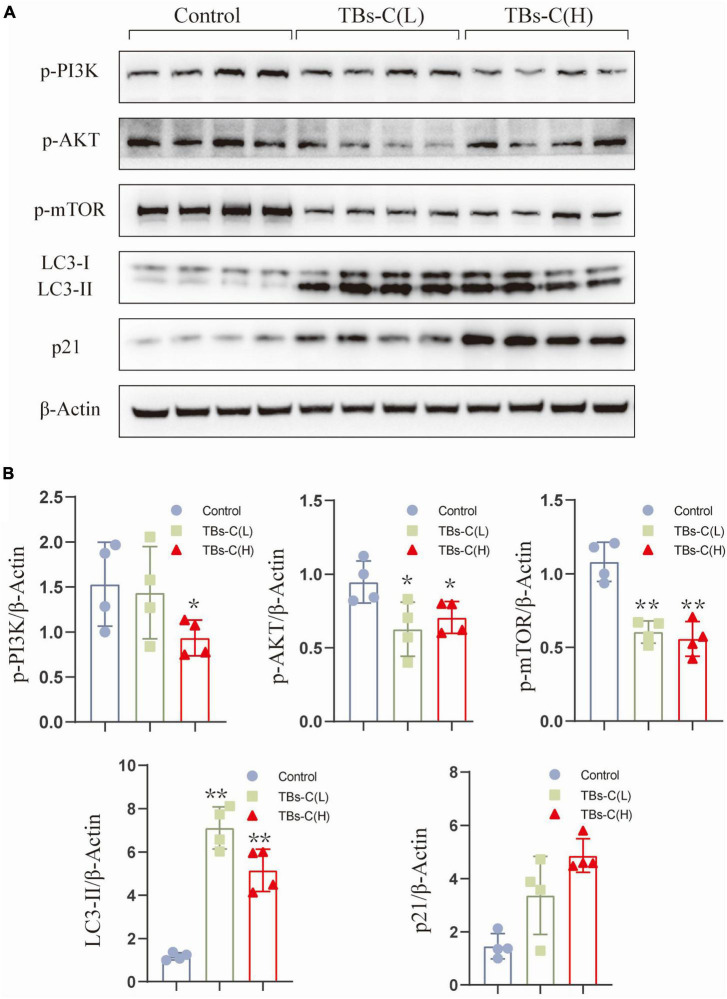
TBs-C inhibits the PI3K/AKT/mTOR pathway *in vitro*. **(A)** The protein was extracted from tumors in each experimental group, and the expression of p-PI3K, p-AKT, p-mTOR, p21, and LC3 were detected by Western blotting. **(B)** Density analysis of Western blotting results among the study groups. TBs-C (L), TBs-C low-dose group, TBs-C (H), TBs-C high-dose group, *p < 0.05, **p < 0.01.

To elucidate the mechanism of TBs-C *in vitro*, tumor protein was extracted from four mice that were randomly selected from the three groups for western blot detection. As illustrated in Figures 9A,B, the levels of p-PI3K, p-Akt, and p-mTOR were significantly lower in xenograft tumors treated with low- or high-dose TBs-c than in the control group (*p* < 0.05). Additionally, the expression of p21, which is downstream of AKT, was found to be elevated in the treatment groups, which agreed with the *in vitro* findings. Furthermore, the autophagy marker LC3-II was upregulated in the TBs-C treatment groups, thus denoting enhanced autophagy in the tumors. In summary, TBs-C induces autophagy by inhibiting the PI3K/AKT/mTOR pathway and augmenting p21 expression via the AKT/p21 signaling axis *in vitro*.

## Discussion

Tea polyphenols are the key biologically active ingredients in green tea. Their antitumor properties have been demonstrated in many tumor cell types (31, 37). In this study, the antilung cancer effects of TP and TBs-C were compared. The results (Figure 1) indicated that the IC50 values of TBs-C were significantly lower than those of TP in several lung cancer cells (*p* < 0.05), which alluded that TBs-C may exhibit a stronger antitumor activity on human NSCLC cells than TP. Recent research has shown that TBs also exhibit antiproliferative and apoptosis-inducing effects against lung cancer (38, 39). In the present study, it was confirmed that TBs-C suppressed proliferation, induced G1 arrest, and accelerated apoptosis in A549 and H2030 cells with the aid of CCK-8, colony formation, flow cytometry, and annexin V/7AAD assays (Figure 2).

The role of autophagy in the occurrence and development of malignant tumors has garnered much attention in recent years (11, 32). The process plays a dual role in tumorigenesis (28); however, the relationship between TBs and autophagy has not been reported so far. In the published studies on the effect of tea extract on autophagy, Izdebska et al. showed that green tea extract induces protective autophagy in A549 cells (40). EGCG-induced autophagy in cancer mostly leads to cell death (14, 41). First, the results of western blot and CYTO-ID green detection reagent assays asserted that TBs-C induces the accumulation of autophagosomes in NSCLC cells (Figure 3). The autophagy downstream inhibitor Baf A1 and the tandem sensor RFP-GFP-LC3B were also used to confirm that TBs-C treatment promoted the autophagic flux in lung cancer cells (Figure 4). In this study, the occurrence of autophagy-mediated cell death was confirmed using the autophagy inhibitor 3-MA and the specific small interfering RNA (siRNA) targeting ATG5. Under these conditions, the antitumor effect of TBs-C was partially inhibited in NSCLC cells (Figure 5). These findings strongly suggest that TBs-C suppresses the proliferation of lung cancer cells and promotes their death by enhancing the autophagic flux.

The PI3K/AKT/mTOR signaling pathway is overactive in many cancers and is strongly linked to cell proliferation, cell cycle, autophagy, and apoptosis (34). The PI3K-AKT-mTOR pathway has been identified to be a potential therapeutic target in NSCLC. Several inhibitors, including sirolimus (an mTOR inhibitor) and perifosine (a dual PI3K/AKT inhibitor), have entered the lung cancer clinical trial stage (42). Fu et al. observed that TBs downregulate p-AKT by upregulating Bax (43). The results displayed in Figure 6 suggest that TBs-C inhibits the activation of the PI3K/AKT/mTOR pathway in human NSCLC cells. To establish whether this mechanism is mainly involved in TBs-C-enhanced autophagy, the cells were cotreated with TBS-C and SC79, a phosphorylated AKT activator. The results signified that cotreatment with SC79 partially reversed TBs-C-induced inhibition of the PI3K/AKT/mTOR pathway. Furthermore, the effects of TBs-C on autophagy and cell death in lung cancer cells were reversed (Figure 7). These findings indicate that TBs-C chiefly enhances autophagy in lung cancer cells via the PI3K/AKT/mTOR pathway.

Previous studies have shown that TBs can cause cell cycle arrest (39, 43), but the underlying mechanism is yet to be elucidated. The AKT/p21 signaling axis is crucial for cell cycle regulation. The decreased phosphorylation of AKT results in higher p21 expression, which can in turn lead to G1 phase cell cycle arrest (36). Previous research has shown that the suppression of the PI3K/AKT/mTOR pathway results in cell cycle arrest in the G1 phase. The possible underlying mechanism is the downregulation of mTOR, which blocks cyclin D from binding to CDK4/6, thereby preventing the transition from G1 to S phase (44). After treatment with TBs-C, phosphorylated AKT and mTOR were downregulated, leading to increased p21 expression and G1 phase arrest in the lung cancer cells (Figure 6). In the rescue experiment, cotreatment with SC79 reduced p21 expression and partially restored the cell cycle arrest induced by TBs-C (Figure 7). These findings imply that TBs-C causes G1 phase arrest in lung cancer cells by affecting the PI3K/AKT/mTOR pathway and the AKT/p21 signaling axis.

Natural plant extracts are in high demand because they are safe and cause few side effects when used in cancer prevention or therapy (45). Studies have suggested that TBs exhibit relatively low toxicity in animal experiments (39, 46, 47). In this study too, TBs-C inhibited tumor growth in A549 xenograft tumors in nude mice without causing cardiac, brain, or kidney toxicity (Figures 8, 9).

**FIGURE 10:**
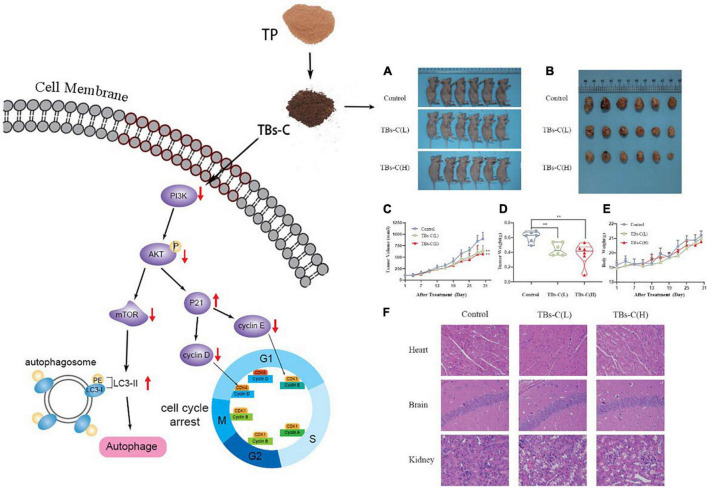
Schematic diagram of TBs-C inhibiting human NSCLC cells *in vitro* and *in vivo*. *In vitro*, TBs-C is capable of increasing autophagic flux, inhibiting cell proliferation, enhancing apoptosis, and inducing G1 cell cycle arrest via the PI3K/AKT/mTOR pathway and AKT/p21 axis. *In vivo*, TBs-C enhances autophagy and inhibits tumor growth by downregulating the PI3K/AKT/mTOR pathway without causing cardiac, brain, or kidney toxicity.

In conclusion, TBs-C displays a superior antitumor effect on NSCLC cells when compared with TP. *In vitro*, TBs-C is capable of increasing autophagic flux, inhibiting cell proliferation, enhancing apoptosis, and inducing G1 cell cycle arrest via inhibition of the PI3K/AKT/mTOR pathway. *In vivo*, TBs-C enhances autophagy and inhibits tumor growth by downregulating the PI3K/AKT/mTOR pathway with low toxicity (Figure 10). These findings are likely to aid in the advancement of research and in the commercialization of chemically prepared TBs.

## Data Availability Statement

The raw data supporting the conclusions of this article will be made available by the authors, without undue reservation.

## Ethics Statement

The animal study was reviewed and approved by the Animal Care Committee of Tongji Hospital, Huazhong University of Science and Technology.

## Author Contributions

XC: conceptualization, methodology, supervision, and writing—review and editing. YW and NZ: investigation. YY, CW, and BW: data curation. YY, CW, and WZ: validation. YW: writing—original draft preparation. NZ and XC: resources. All authors have read and agreed to the published version of the manuscript.

## Conflict of Interest

The authors declare that the research was conducted in the absence of any commercial or financial relationships that could be construed as a potential conflict of interest.

## Publisher’s Note

All claims expressed in this article are solely those of the authors and do not necessarily represent those of their affiliated organizations, or those of the publisher, the editors and the reviewers. Any product that may be evaluated in this article, or claim that may be made by its manufacturer, is not guaranteed or endorsed by the publisher.
